# Short-term cryoprotectant-free cryopreservation at −20°C does not affect the viability and regenerative capacity of nanofat

**DOI:** 10.3389/fbioe.2024.1427232

**Published:** 2024-07-01

**Authors:** Ettore Limido, Andrea Weinzierl, Emmanuel Ampofo, Yves Harder, Michael D. Menger, Matthias W. Laschke

**Affiliations:** ^1^ Institute for Clinical and Experimental Surgery, Saarland University, Homburg, Germany; ^2^ Department of Plastic Surgery and Hand Surgery, University Hospital Zurich, Zurich, Switzerland; ^3^ Department of Plastic, Reconstructive and Aesthetic Surgery, Ospedale Regionale di Lugano, Ente Ospedaliero Cantonale (EOC), Lugano, Switzerland; ^4^ Faculty of Biomedical Sciences, Università della Svizzera Italiana, Lugano, Switzerland

**Keywords:** nanofat, cryopreservation, wound healing, platelet-rich plasma, vascularization, angiogenesis

## Abstract

Nanofat is an autologous fat derivative with high regenerative activity, which is usually administered immediately after its generation by mechanical emulsification of adipose tissue. For its potential repeated use over longer time, we herein tested whether cryopreservation of nanofat is feasible. For this purpose, the inguinal fat pads of donor mice were processed to nanofat, which was i) frozen and stored in a freezer at −20°C, ii) shock frozen in liquid nitrogen with subsequent storage at −80°C or iii) gradually frozen and stored at −80°C. After 7 days, the cryopreserved nanofat samples were thawed and immunohistochemically compared with freshly generated nanofat (control). Nanofat frozen and stored at −20°C exhibited the lowest apoptotic rate and highest densities of blood and lymph vessels, which were comparable to those of control. Accordingly, nanofat cryopreserved at −20°C or control nanofat were subsequently fixed with platelet-rich plasma in full-thickness skin defects within dorsal skinfold chambers of recipient mice to assess vascularization, formation of granulation tissue and wound closure by means of stereomicroscopy, intravital fluorescence microscopy, histology and immunohistochemistry over 14 days. These analyses revealed no marked differences between the healing capacity of wounds filled with cryopreserved or control nanofat. Therefore, it can be concluded that cryopreservation of nanofat is simply feasible without affecting its viability and regenerative potential. This may broaden the range of future nanofat applications, which would particularly benefit from repeated administration of this autologous biological product.

## 1 Introduction

Nanofat is generated by mechanical emulsification and filtration of adipose tissue and has been evaluated in various experimental and clinical settings to prove its beneficial regenerative properties (Menkes et al., 2020; Qi et al., 2021; Rageh et al., 2021; Han et al., 2022; Schilling et al., 2023; Tran et al., 2023). This fat derivative contains high quantities of growth factors, mesenchymal stem cells and microvascular fragments, which stimulate blood vessel and tissue formation (Jeyaraman et al., 2021). Accordingly, nanofat has been shown to improve the outcome of scar treatment, androgenic alopecia and rejuvenation of the face (Uyulmaz et al., 2018; Surowiecka and Struzyun, 2022; La Padula et al., 2023). Moreover, we have recently analyzed the effects of nanofat on the healing of full-thickness skin wounds in a mouse dorsal skinfold chamber model (Limido et al., 2024). For this purpose, we incorporated nanofat in activated platelet-rich plasma (PRP) gel to guarantee its stable fixation in the wound bed. Of interest, we found that PRP + nanofat-treated wounds exhibited an improved vascularization, lymphatic drainage and healing when compared to PRP-treated and empty control wounds (Limido et al., 2024).

Wound healing associated with systemic comorbidities or large tissue defects often requires a long time and, thus, repeated wound treatments (Marston and Dermagraft Diabetic Foot Ulcer Study Group, 2006; Anderson and Hamm, 2014; Sen, 2021). Therefore, the use of autologous nanofat to improve the healing of such complex and chronic wounds may require multiple surgical fat harvesting procedures, which is laborious and not a realistically feasible option in clinical routine. On the other hand, many patients undergo liposuction for the removal of large amounts of adipose tissue that is subsequently just discarded, although it may be highly useful for later nanofat applications in these patients due to future morbidities. To address these issues, cryopreservation of nanofat may represent an attractive approach.

Cryopreservation of cells and tissues including fat derivatives has shown promising results (Laschke et al., 2003; Xu et al., 2012; Laschke et al., 2018; Tao et al., 2023). Over the years, various cryopreservation protocols have been established that involve different freezing procedures and storage temperatures (MacRae et al., 2004; Villaverde-Doménech et al., 2021; Tao et al., 2023). Many of them use cryoprotective agents, such as ethylene glycol, glycerol, methanol or dimethyl sulfoxide (DMSO), to prevent ice crystal formation by interfering with hydrogen bonding between water molecules (Karlsson et al., 1994; Pegg, 2007; Best, 2015; Whaley et al., 2021). Of note, these agents also exert cytotoxic effects on different cell components, including chromosomes, mitochondria and membranes (Best, 2015). Accordingly, they should be completely removed without leaving any remains after cryopreservation. However, this is not possible in case of liquid nanofat without markedly affecting its original heterogeneous mixture of growth factors, microvascular fragments, cells and extracellular matrix components.

Therefore, the aim of the present study was to develop a simple, cryoprotectant-free cryopreservation protocol for the storage of nanofat. For this purpose, we first tested *ex vivo* the effects of different cooling procedures and storage temperatures on the viability and vessel content of nanofat samples. In addition, we analyzed the *in vivo* regenerative capacity of cryopreserved nanofat in comparison to freshly generated nanofat in a murine wound healing model, as performed previously (Limido et al., 2024).

## 2 Materials and methods

### 2.1 Animals

All animal experiments were conducted in accordance with the European legislation on animal care (Directive 2010/63/EU) and the NIH Guidelines for the Care and Use of Laboratory Animals (NIH publication #85-23 Rev. 1985). They were approved by the local governmental authorities (permission number: 06/2022).

For *ex vivo* analyses, C57BL/6J wildtype mice (Institute for Clinical and Experimental Surgery, Saarland University, Homburg, Germany) with a mean age of 6 months and body weight of 26 g served as donors for inguinal fat tissue harvesting and nanofat generation. For *in vivo* experiments, C57BL/6J wildtype mice with a mean age of 8 months and body weight of 25 g served as blood donors for the generation of PRP. In addition, green fluorescent protein (GFP)^+^ mice (C57BL/6-Tg (CAG-EGFP)1Osb/J; The Jackson Laboratory, Bar Harbor, ME, United States) with a mean age of 6 months and body weight of >30 g were used as fat tissue donors. C57BL/6J wildtype mice with a mean age of 4 months and a body weight of 25 g were equipped with dorsal skinfold chambers. They were housed in individual cages to prevent mutual injuries due to the chambers. All animals had free access to standard pellet food (Altromin, Lage, Germany) and tap water.

### 2.2 Anesthesia

All procedures, including inguinal fat harvesting, preparation of the dorsal skinfold chamber and repeated microscopies, were performed in general anesthesia by means of an intraperitoneal (i.p.) injection of ketamine (100 mg/kg body weight; Ursotamin^®^; Serumwerke Bernburg, Bernburg, Germany) and xylazine (12 mg/kg body weight; Rompun^®^; Bayer, Leverkusen, Germany). For post-operative pain management 10 mg/kg of carprofen (Rimadyl^®^; Zoetis Deutschland GmbH, Berlin, Germany) was injected subcutaneously during the preparation of the dorsal skinfold chamber.

### 2.3 Generation of PRP and nanofat

PRP and nanofat were generated according to standard procedures, as previously described in detail (Weinzierl et al., 2022). Briefly, anesthetized donor mice were fixed in supine position on a heating pad and a median laparotomy was performed with subsequent puncture of the vena cava inferior for blood sampling. The harvested blood was then centrifuged to generate PRP, which was stored at −20°C for further use. To generate nanofat, the inguinal fat pads of donor mice were harvested, minced and mechanically processed to nanofat via repeated shuffle and filtration steps (Weinzierl et al., 2022).

### 2.4 Cryopreservation of nanofat

In the present study, three different methods for the cryopreservation of nanofat were tested, whereas freshly isolated nanofat served as control. For this purpose, the nanofat from each donor mouse (n = 4) was equally divided in four separated plastic monovettes (Sarstedt, Nümbrecht, Germany). The first sample (fresh nanofat; control) was then immediately embedded in agarose and fixed in 4% formalin for further histological and immunohistochemical analyses. The second sample (−20°C) was put in a freezer with a constant temperature of −20°C. The third sample (SF −80°C) was shock frozen in liquid nitrogen at −196°C and subsequently stored in a freezer at −80°C. The fourth sample (GF −80°C) was gradually frozen (1°C/min) in a Nalgene cryo-freezing container (Fisher Scientific, Schwerte, Germany) until −80°C. After 7 days, the cryopreserved samples were thawed by incubating the monovettes in a water bath at a constant temperature of 37°C. Thereafter, they were also further processed for histological and immunohistochemical analyses.

### 2.5 Dorsal skinfold chamber model

The effects of cryopreserved and freshly isolated nanofat from 8 GFP^+^ donor mice on wound healing were analyzed in a well-established dorsal skinfold chamber model (Limido et al., 2024) (Supplementary Figure S1). For this purpose, two symmetrical titanium frames with a central observation window (Irola Industriekomponenten GmbH & Co. KG, Schonach, Germany) were implanted onto the back of 16 C57BL/6J mice, as described previously (Laschke and Menger, 2016). After 2 days, a full-thickness skin defect (diameter: 4 mm) was created within the observation window using a biopsy punch (GSK Consumer Healthcare, GMDT, Clocherane, Youghal Road, Dungarvan, Co. Waterford, Ireland) and micro-scissors (Fine Science Tools GmbH, Germany). The wounds were filled with a suspension of 3 µL freshly generated nanofat (control; n = 8) or cryopreserved nanofat (cryo; n = 8) in 5 µL PRP. The PRP was activated with 2 µL thrombin (10 U/mL dissolved in 10% CaCl_2_; Sigma-Aldrich, Taufkirchen, Germany) to transform it into a stable gel and, thus, to fix the nanofat inside the wound bed (Limido et al., 2024). Thereafter, the observation window of the chamber was closed by means of a cover slip and a snap ring to protect the wounds from external manipulation by the animals.

### 2.6 Stereomicroscopy

The size of the wounds was repeatedly analyzed over time using stereomicroscopy on days 0 (day of wound creation), 3, 6, 10 and 14. For this purpose, the anesthetized mice equipped with dorsal skinfold chambers were fixed onto a plexiglass stage and the observation window with the wounds inside was placed horizontally under a stereomicroscope (Leica M651, Wetzlar, Germany). Images were captured using a camera and DVD recording system and subsequently analyzed offline with the analysis system CapImage (version 8.10.1; Zeintl, Heidelberg, Germany). The wound area was measured at all time points and expressed in % of the wound area on day 0.

### 2.7 Intravital fluorescence microscopy

Directly after stereomicroscopy, the vascularization of the wounds was analyzed by intravital fluorescence microscopy. For contrast enhancement, 100 µL of the blood plasma marker 5% fluorescein isothiocyanate (FITC)-labeled dextran (150,000 Da; Sigma-Aldrich) was intravenously injected into the retrobulbar plexus. The dorsal skinfold chamber of the animals was then positioned under a fluorescence epi-illumination microscope (Axiocam 702 mono; Zeiss, Oberkochen, Germany). Microscopic images were recorded on an external hard drive (Intenso International GmbH, Germany) using ZEN software (version 3.7; Zeiss) for further quantitative analysis by means of CapImage (version 8.10.1; Zeintl). This analysis included the determination of the total number of perfused regions of interest (ROIs, %), the functional microvessel density (cm/cm^2^) as well as the diameter (µm), centerline red blood cell (RBC) velocity (µm/s), shear rate (s^−1^) and volumetric blood flow (pL/s) of individual microvessels, as described previously (Limido et al., 2024). At the end of the *in vivo* experiments on day 14, the animals were sacrificed by cervical dislocation and the wounds with the surrounding tissue were harvested for further analyses by means of histology and immunohistochemistry.

### 2.8 Histology and immunohistochemistry

Tissue samples were fixed in 4% formalin, embedded in paraffin and cut in 3-µm sections. Hematoxylin and eosin (HE) staining was performed according to standard protocols. For light and immunofluorescent analyses, additional sections were stained with primary antibodies against cleaved caspase-3 (casp-3; 1:100; Cell Signaling; Danvers, MA, United States), CD31 (1:100; dianova GmbH, Hamburg, Germany), lymphatic vessel endothelial hyaluronan receptor (LYVE)-1 (1:200; Abcam, Cambridge, United Kingdom), GFP (1:100; Rockland, Limerik, PA, United States), collagen (Col) I (1:250; Abcam), Col III (1:100; Proteintech, Rosemont, IL, United States), CD86 (1:100; Cell Signaling) and CD163 (1:100; Proteintech). A horseradish peroxidase-labeled goat anti-rabbit IgG antibody (1:200; dianova GmbH), a goat-anti-rat IgG-Alexa555 antibody (1:100; Molecular Probes, Eugene, OR, United States) and a goat-anti-rabbit IgG-Alexa555 antibody (1:200; Molecular Probes) served as secondary antibodies. 3-Amino-9-ethylcarbazole (Abcam, Cambridge, United Kingdom) was used as chromogen and cell nuclei were stained with Hoechst 33342 (2 μg/mL: Sigma-Aldrich) on immunofluorescence sections.

The stained sections were quantitatively analyzed by means of a BX53 microscope and the imaging software cellSens Dimension (version 1.11; Olympus, Hamburg, Germany). This analysis included the assessment of the following parameters: Casp-3^+^ cells/all cells (%), microvessel density (mm^−2^), lymph vessel density (mm^−2^), epithelialization (%), granulation tissue formation (%), cellular density (mm^−2^), total Col I and Col III ratio (wound/skin), CD31^+^/GFP^+^ microvessels (%), LYVE-1^+^/GFP^+^ lymph vessels (%), CD86^+^ M1 and CD163^+^ M2 macrophages (mm^−2^) as well as M2/M1 ratio (Limido et al., 2024).

### 2.9 Statistical analysis

All data sets were first tested for normal distribution and equal variance (GraphPad Prism version 10.1.2; GraphPad Software, San Diego, CA, United States). Differences between two groups were analyzed by an unpaired Student’s *t*-test (parametric data) or a Mann-Whitney rank sum test (non-parametric data). Differences between more than two groups were assessed by one-way ANOVA (parametric data) or ANOVA on ranks (non-parametric data) followed by a Student-Newman-Keuls *post hoc* test. All values are expressed as means ± standard error of the mean (SEM). Statistical significance was accepted for values of *p* < 0.05.

## 3 Results

### 3.1 Cryopreservation and characterization of nanofat

In a first set of experiments, three different methods for the cryopreservation of nanofat were tested. These included i) simple freezing and storage in a freezer at a constant temperature of −20°C, ii) shock freezing in liquid nitrogen with subsequent storage at −80°C and iii) gradual freezing (1°C/min) and storage at −80°C. After 7 days, the cryopreserved nanofat samples were thawed in a water bath at a constant temperature of 37°C and compared to freshly generated nanofat, which served as control.

**FIGURE 1:**
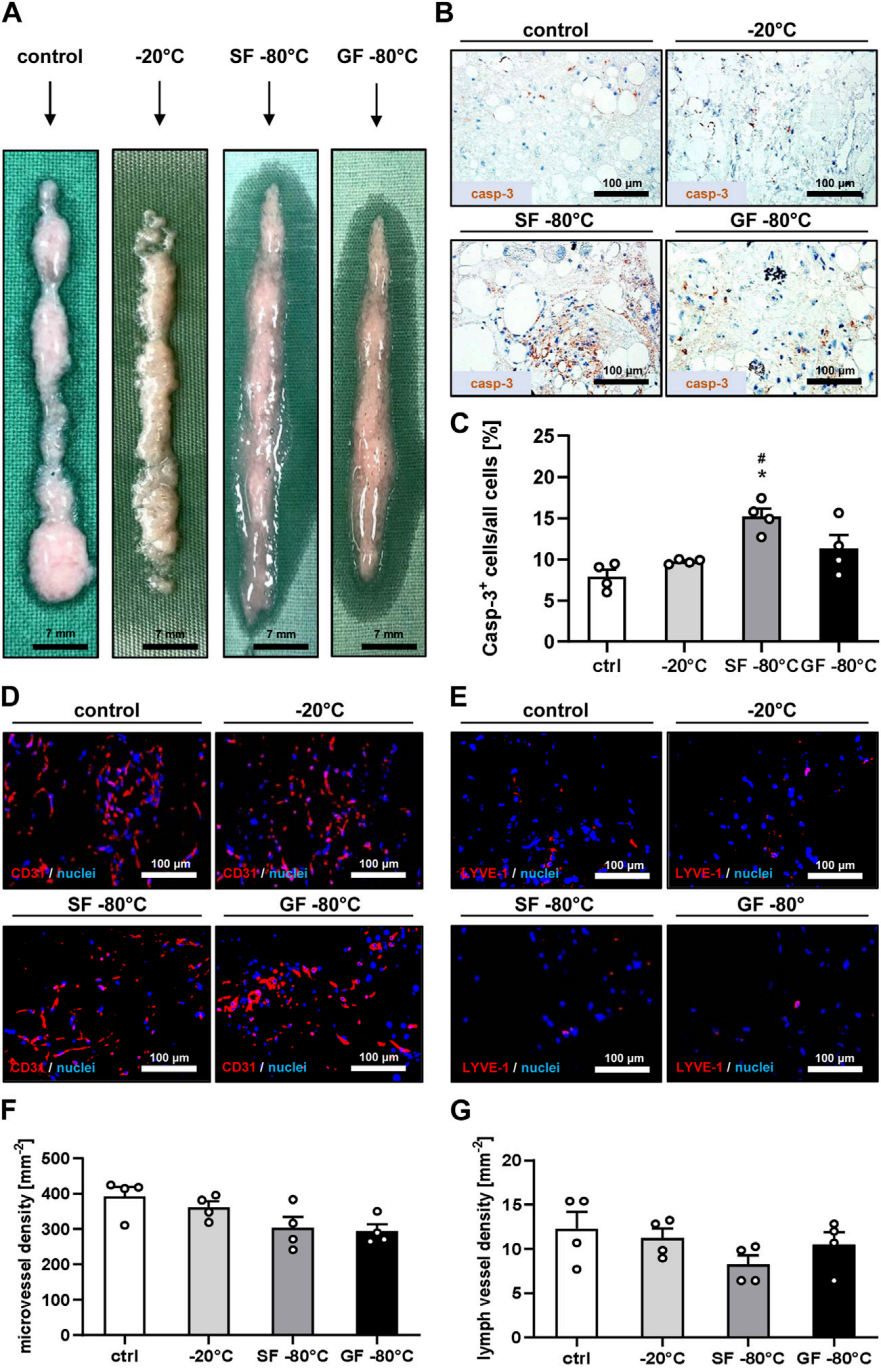
Cryopreservation and characterization of nanofat. **(A)** Macroscopic appearance of fresh nanofat (control) as well as nanofat that was frozen and stored at −20°C, shock frozen (SF) in liquid nitrogen or gradually frozen (GF) with subsequent storage at −80°C. **(B, C)** Immunohistochemical detection of casp-3^+^ apoptotic cells **(B)** and their quantitative analysis **(C)** in fresh nanofat (control; white bar; n = 4) as well as nanofat that was frozen and stored at −20°C (light gray bar; n = 4), shock frozen (SF) in liquid nitrogen (dark gray bar; n = 4) or gradually frozen (GF) with subsequent storage at −80°C (black bar; n = 4). Means ± SEM; *p < 0.05 vs control; ^#^ *p* < 0.05 vs. −20°C. **(D–G)** Immunohistochemical detection of CD31^+^ microvessels **(D)** and LYVE-1^+^ lymph vessels **(E)** and their quantitative analysis **(F, G)** in fresh nanofat (control; white bars; n = 4) as well as nanofat that was frozen and stored at −20°C (light gray bars; n = 4), shock frozen (SF) in liquid nitrogen (dark gray bars; n = 4) or gradually frozen (GF) with subsequent storage at −80°C (black bars; n = 4). Means ± SEM. No significant differences between the groups.

Macroscopically, no differences between the four sample types were detected. They exhibited a white to reddish coloration (Figure 1A). Immunohistochemical analyses showed the lowest rate of apoptotic cells in freshly generated nanofat and nanofat samples that were stored at −20°C (Figures 1B, C). The highest rate of apoptotic cell death was found in nanofat samples that were shock frozen and stored at −80°C (Figures 1B, C). In line with these findings we also detected the highest densities of blood and lymph vessels in control and −20°C samples, which, however, did not significantly differ from those in samples that were stored at −80°C (Figures 1D–G). Based on these results we selected the nanofat samples that were cryopreserved at −20°C for subsequent *in vivo* wound healing experiments with freshly generated nanofat serving as control.

### 3.2 *In vivo* microscopy of healing wounds

**FIGURE 2:**
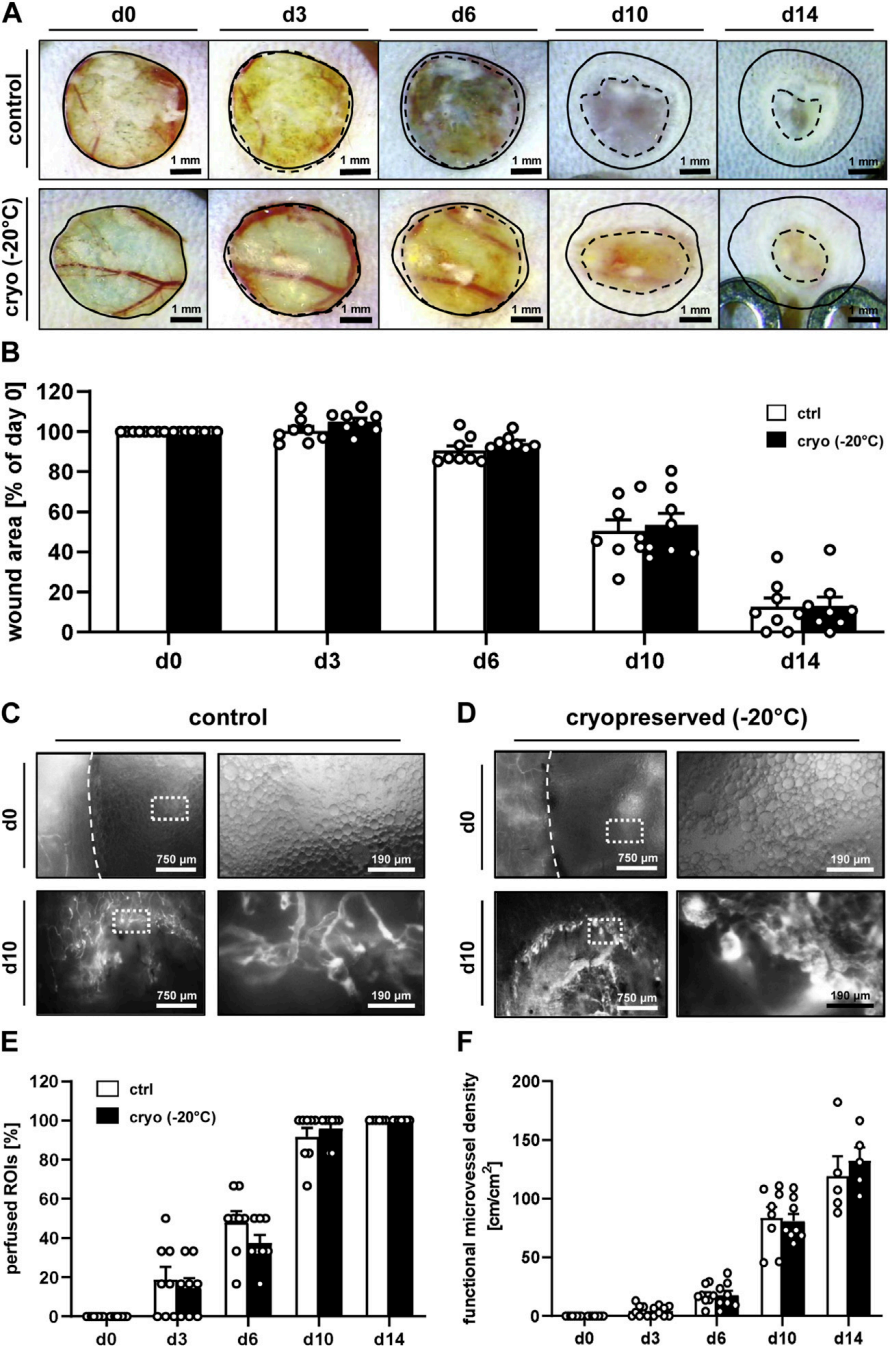
*In vivo* microscopy of healing wounds. **(A)** Stereomicroscopy of wounds filled with fresh (control) or cryopreserved nanofat on days 0, 3, 6, 10 and 14 (initial wound borders = closed lines; wound borders at the indicated time points = broken lines). **(B)** Wound area (% of day 0) of wounds filled with fresh (white bars; n = 8) or cryopreserved (black bars; n = 8) nanofat on days 0, 3, 6, 10 and 14, as assessed by stereomicroscopy. Means ± SEM. No significant differences between the groups. **(C, D)** Intravital fluorescence microscopy of wounds filled with fresh [control, **(C)**] or cryopreserved **(D)** nanofat on days 0 and 10. Higher magnification of dotted frames is shown in the right panels. **(E, F)** Perfused ROIs [**(E)**, %)] and functional microvessel density [**(F)**, cm/cm^2^] of wounds filled with fresh (white bars; n = 8) or cryopreserved (black bars; n = 8) nanofat on days 0, 3, 6, 10 and 14, as assessed by intravital fluorescence microscopy. Means ± SEM. No significant differences between the groups.

The used dorsal skinfold chamber model enabled a repeated *in vivo* analysis of the wound healing process by means of stereomicroscopy and intravital fluorescence microscopy throughout an observation period of 14 days (Figures 2A–F). Stereomicroscopy of the wounds at the beginning of the analysis proved a comparable initial wound size in the control and cryopreservation group, which nearly remained constant until day 6 (Figures 2A, B). Thereafter, wound closure rapidly progressed until day 14 without significant differences between the two groups (Figures 2A, B).

Intravital fluorescence microscopy revealed a comparable vascularization of wounds that were filled with either freshly generated or cryopreserved nanofat (Figures 2C–F). In fact, in both groups no blood-perfused microvessels could be detected inside the wounds on day 0, though the nanofat was clearly visible due to its oil droplets (Figures 2C, D). Throughout the following days, blood-perfused microvessels progressively grew from the border towards the center of the wounds, which finally exhibited a complete vascularization on day 14, as indicated by 100% perfused ROIs with a functional microvessel density of ∼130 cm/cm^2^ in both groups (Figures 2E, F). Of note, at this late time point epithelialization of some wounds was already well advanced, which prevented their analysis by means of intravital fluorescence microscopy. The additional assessment of microhemodynamic parameters further showed no significant differences of the microvessels’ diameter, centerline RBC velocity, shear rate and volumetric blood flow in wounds filled with freshly generated or cryopreserved nanofat (Supplementary Table S1).

### 3.3 Histology and immunohistochemistry of healing wounds

**FIGURE 3:**
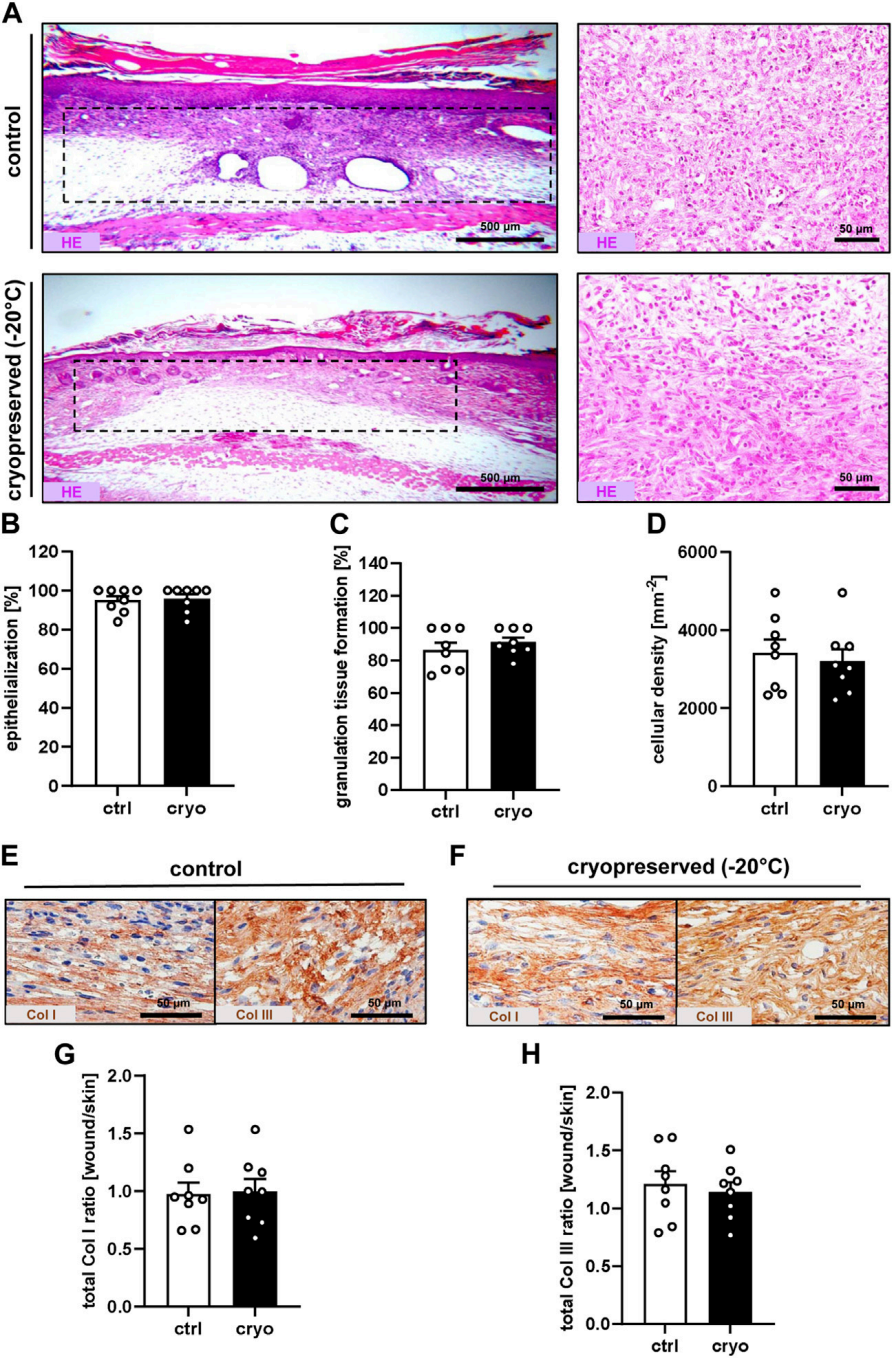
Tissue and extracellular matrix formation in healing wounds. **(A)** HE-stained sections (left panels = overview; right panels = higher magnification) of wounds (borders marked by broken lines) filled with fresh (control) or cryopreserved nanofat on day 14. **(B–D)** Epithelialization [**(B)**, %], granulation tissue formation [**(C)**, %] and cellular density [(D), mm^−2^] of wounds filled with fresh (white bars; n = 8) or cryopreserved (black bars; n = 8) nanofat on day 14, as assessed by histology. Means ± SEM. No significant differences between the groups. **(E,F)** Immunohistochemical detection of Col I and III in wounds filled with fresh [control, **(E)**] or cryopreserved **(F)** nanofat on day 14. **(G,H)** Total Col I **(G)** and Col III **(H)** ratio (wound/skin) of wounds filled with fresh (white bars; n = 8) or cryopreserved (black bars; n = 8) nanofat on day 14, as assessed by immunohistochemistry. Means ± SEM. No significant differences between the groups.

At the end of the *in vivo* observation period on day 14, the healing wounds were additionally evaluated by means of histology and immunohistochemistry. The analysis of HE-stained sections revealed a comparable epithelialization, granulation tissue formation and cellular density of wounds filled with freshly generated or cryopreserved nanofat (Figures 3A–D). Immunohistochemical analyses further showed that they did not significantly differ in their Col I and Col III content (Figures 3E–H).

**FIGURE 4:**
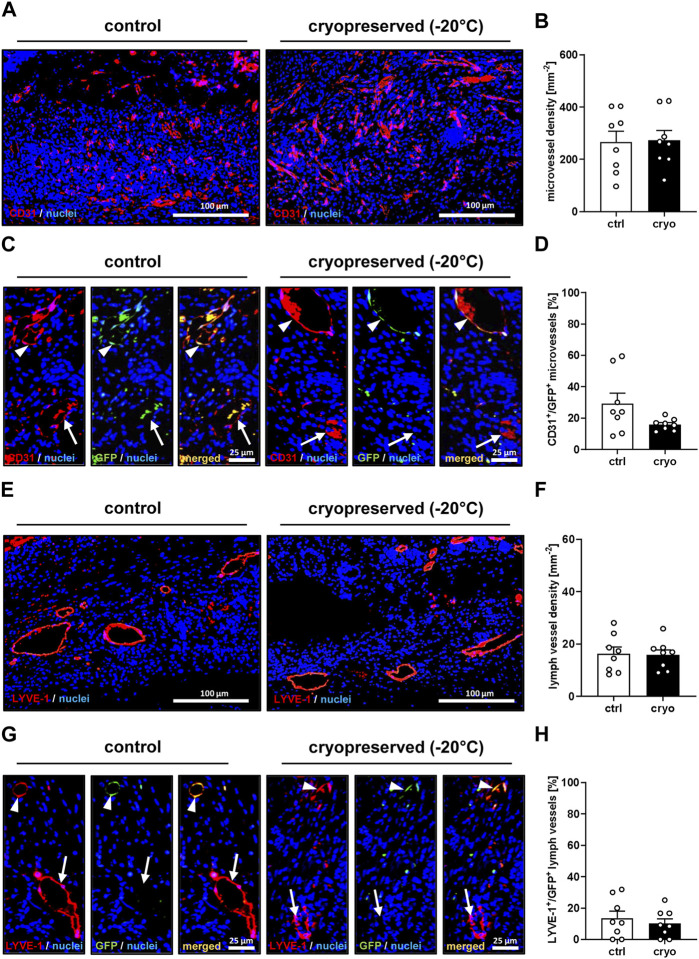
Vascularization and lymphatic drainage of healing wounds. **(A)** Immunohistochemical detection of CD31^+^ microvessels in wounds filled with fresh (control) or cryopreserved nanofat on day 14. **(B)** Microvessel density (mm^−2^) of wounds filled with fresh (white bars; n = 8) or cryopreserved (black bars; n = 8) nanofat on day 14, as assessed by immunohistochemistry. Means ± SEM. No significant differences between the groups. **(C)** Immunohistochemical detection of CD31^+^/GFP^−^ (arrows) and CD31^+^/GFP^+^ (arrowheads) microvessels in wounds filled with fresh (control) or cryopreserved nanofat on day 14. **(D)** CD31^+^/GFP^+^ microvessels (%) in wounds filled with fresh (white bars; n = 8) or cryopreserved (black bars; n = 8) nanofat on day 14, as assessed by immunohistochemistry. Means ± SEM. No significant differences between the groups. **(E)** Immunohistochemical detection of LYVE-1^+^ lymph vessels in wounds filled with fresh (control) or cryopreserved nanofat on day 14. **(F)** Lymph vessel density (mm^−2^) of wounds filled with fresh (white bars; n = 8) or cryopreserved (black bars; n = 8) nanofat on day 14, as assessed by immunohistochemistry. Means ± SEM. No significant differences between the groups. **(G)** Immunohistochemical detection of LYVE-1^+^/GFP^−^ (arrows) and LYVE-1^+^/GFP^+^ (arrowheads) lymph vessels in wounds filled with fresh (control) or cryopreserved nanofat on day 14. **(H)** LYVE-1^+^/GFP^+^ microvessels (%) in wounds filled with fresh (white bars; n = 8) or cryopreserved (black bars; n = 8) nanofat on day 14, as assessed by immunohistochemistry. Means ± SEM. No significant differences between the groups.

Moreover, they also exhibited a comparable density of blood and lymph vessels (Figures 4A, B, E, F). Of interest, in both groups a significant number of these vessels were GFP^+^, indicating their direct origin from the nanofat of GFP^+^ donor animals (Figures 4C, D, G, H).

Finally, CD86 and CD163 stainings were performed to quantify the number of M1 and M2 polarized macrophages inside the wounds (Supplementary Figures S2A–E). The numbers of both macrophage phenotypes showed a tendency towards lower values in wounds filled with cryopreserved nanofat (Supplementary Figures S2A–D). However, the M2/M1 ratio did not significantly differ between the two groups (Supplementary Figure S2E).

## 4 Discussion

In the present study, we could demonstrate that cryopreservation of nanofat does not affect its viability and *in vivo* regenerative capacity. From a clinical point of view, this finding may be of particular relevance in the management of chronic wounds, which do not heal after a single treatment but require several interventions for adequate closure. In this case, the repeated application of autologous cryopreserved nanofat would be a feasible approach without the necessity of multiple sessions of fat harvesting for the patient.

Nanofat results from the mechanical emulsification of adipose tissue. Accordingly, it represents a heterologous mixture of cells, blood and lymphatic vessel fragments, extracellular matrix components as well as soluble growth factors and cytokines (Jeyaraman et al., 2021). Hence, nanofat effectively stimulates regenerative processes in a physiological manner using the body’s own resources similar to other natural biological products, such as hypoxia-preconditioned secretomes (Laschke and Menger, 2022). Moreover, in contrast to complex and time-consuming procedures involving intensive cell manipulation, the generation of nanofat is rapid and simple, which enables its use in clinical practice much easier in terms of safety requirements and approval restrictions. On the other hand, the composition of nanofat and, thus, its regenerative properties may markedly differ between individual patients, as it has previously been described for PRP (Lang et al., 2018). Another major challenge is the cryopreservation of nanofat, because it cannot be simply mixed with cryoprotectants, whose residue-free removal by centrifugation and washing steps after the thawing process would markedly change its composition. Therefore, we herein chose a cryoprotectant-free protocol, as it has previously been applied for fat grafts and other fat derivatives (MacRae et al., 2004; Tao et al., 2023).

Besides the decision of whether or not to use cryoprotectants, the freezing kinetics and temperatures are also of utmost importance for the success of cryopreservation. To address this issue, three different types of nanofat cryopreservation were compared. These included i) simple freezing and storage in a freezer at a constant temperature of −20°C, ii) shock freezing in liquid nitrogen with subsequent storage at −80°C and iii) gradual freezing (1°C/min) and storage at −80°C. We found that shock freezing of nanofat resulted in the highest rate of apoptotic cells. This result is in line with other studies reporting that a rapid freezing speed at very low temperatures markedly affects cell viability, whereas gradual freezing significantly reduces the degree of apoptotic cell death (Karlsson et al., 1994; Gal and Pu, 2020). However, we further found that gradual freezing even seems not to be necessary when nanofat is simply put in a freezer at −20°C. In fact, this approach resulted in an apoptotic rate, which was comparable to that of freshly generated nanofat. Moreover, we detected highly similar densities of blood and lymph vessels in freshly generated nanofat and nanofat stored at −20°C. Therefore, we suggest that this cryopreservation method is suitable for nanofat and in addition would be easily implementable in clinical practice.

In view of these preliminary conclusions, we further analyzed the *in vivo* regenerative capacity of nanofat that was cryopreserved at −20°C. For this purpose, we chose the identical experimental setting as in a recent study (Limido et al., 2024), because it bears several advantages. The full-thickness circular skin defects within the observation window of the dorsal skinfold chamber are well protected from scratch artifacts or infection by means of a cover slip. Furthermore, they can be easily accessed for microscopic imaging. Accordingly, this approach did not only allow the repeated stereomicroscopic measurement of the wound area, but also the assessment of various microcirculatory parameters by means of intravital fluorescence microscopy. Particularly the latter technique provides detailed insights into the wound healing process, which is crucially dependent on an adequate blood perfusion of the wound bed (Shen et al., 2016; Grambow et al., 2021).

Our *in vivo* results showed a comparable healing capacity of wounds that were filled with freshly generated or cryopreserved nanofat. In fact, the wounds did not show any significant differences in terms of vascularization, tissue formation and extracellular matrix composition. Moreover, in both groups they contained a significant amount of GFP^+^ blood and lymph vessels. This observation confirms our previous finding that nanofat not only promotes the vascularization of wounds by releasing angiogenic growth factors but also by its rich content of vessel segments, which reassemble into new functional networks after transplantation (Limido et al., 2024). A tendency towards lower M1 and M2 macrophage numbers was the only slight difference that we detected in wounds filled with cryopreserved nanofat when compared to controls. This may be due to a reduced long-term viability of these immune cells within the grafted nanofat, because we did not use a cryoprotectant. On the other hand, cryopreservation may have reduced the levels of inflammatory cytokines within the nanofat, resulting in less macrophages migrating into the wound bed from the surrounding tissue. However, since the numbers of both M1 and M2 macrophages were lower in wounds filled with cryopreserved nanofat, the overall M2/M1 ratio was not affected. This result may further explain the comparable healing capacity of wounds filled with fresh and cryopreserved nanofat, because particularly the ratio between regenerative M2 macrophages and inflammatory M1 macrophages has been shown to crucially determine regenerative processes (Song et al., 2023; Amoupour et al., 2024; Zhao et al., 2024).

Finally, it should also be mentioned that this study has some limitations. In fact, we tested our cryopreservation protocols in combination with a relatively short storage period of only 7 days. Because it is known that the storage time can affect cell viability and regenerative properties of different cell types (Kim et al., 2014; Tao et al., 2023), we therefore cannot exclude that longer storage periods would have resulted in other outcomes related to cell viability. Moreover, we herein used PRP to stably fix the nanofat inside the wound bed. However, PRP itself is a rich source of angiogenic growth factors and its angiogenic activity may markedly differ between individual donors (Limido et al., 2023). To overcome the latter problem, we generated PRP from pooled blood samples of donor mice, which was then used for the filling of all wounds. By this, a PRP-induced bias between the healing capacity of wounds filled with freshly generated or cryopreserved nanofat could be excluded.

Taken together, we herein introduce a simple approach for the cryopreservation of nanofat. Our data demonstrate that the storage of nanofat at −20°C in a conventional freezer does not affect its viability and regenerative properties. Because this approach may be easily implemented into clinical routine procedures, it may help to further broaden the range of nanofat applications, particularly in fields, where repeated administration of this autologous biological product is beneficial for the treatment of non-healing and chronic pathologies.

## Data Availability

The raw data supporting the conclusions of this article will be made available by the authors, without undue reservation.

## Supplementary material

Supplementary Figure S1 Experimental design of the present *in vivo* study. GFP^+^ donor mice served as donors for harvesting of inguinal adipose tissue, which was further processed to nanofat. The nanofat was cryopreserved at −20°C for 7 days, whereas freshly generated nanofat served as control. Cryopreserved and fresh nanofat were fixed by means of PRP in full-thickness circular skin defects within the dorsal skinfold chambers of GFP^−^ recipient animals to investigate wound healing by means of repeated *in vivo* stereomicroscopy and intravital fluorescence microscopy as well as final histological and immunohistochemical analyses.

Supplementary Figure S2 Macrophage phenotypes in healing wounds. **(A, B)** Immunohistochemical detection of CD86^+^ M1 and CD163^+^ M2 macrophages in wounds filled with fresh [control, **(A)**] or cryopreserved **(B)** nanofat on day 14. **(C–E)** M1 macrophages [**(C)**, mm^−2^], M2 macrophages [**(D)**, mm^−2^], and M2/M1 ratio in wounds filled with fresh (white bars; n = 8) or cryopreserved (black bars; n = 8) nanofat on day 14, as assessed by immunohistochemistry. Means ± SEM. *p < 0.05 vs control.

Supplementary Table S1 Diameter (µm), centerline RBC velocity (µm/s), shear rate (s^−1^) and volumetric blood flow (pL/s) of microvessels within wounds filled with fresh (control; n = 8) or cryopreserved (n = 8) nanofat on days 0, 3, 6, 10 and 14, as assessed by intravital fluorescence microscopy.
