# Supplementary material for: Short-term cryoprotectant-free cryopreservation at −20°C does not affect the viability and regenerative capacity of nanofat
Source: Front Bioeng Biotechnol. 2024 Jul 1;12:1427232. doi: 10.3389/fbioe.2024.1427232 (PMC11246958; doi:10.3389/fbioe.2024.1427232)
Supplement: Supplementary file 1 [file Table1.docx]

**Supplementary Table S1.**

**d0 d3 d6 d10 d14**

_________________________________________________________________________________________________

***Diameter [µm]:***

control - 19.1 ± 1.3 17.7 ± 0.6 17.9 ± 0.8 15.5 ± 0.7

cryopreserved - 18.1 ± 1.9 19.1 ± 1.1 17.4 ± 0.8 15.5 ± 0.7

***Centerline RBC velocity [µm/s]:***

control - 138.5 ± 39.8 184.1 ± 29.0 182.0 ± 23.2 201.0 ± 21.3

cryopreserved - 119.5 ± 25.4 126.7 ± 13.9 153.9 ± 19.8 182.8 ± 21.2

***Shear rate [s^-1^]:***

control - 67.6 ± 21.9 94.2 ± 15.9 86.1 ± 12.1 94.3 ± 10.3

cryopreserved - 57.9 ± 13.3 57.1 ± 6.1 77.4 ± 10.8 103.1 ± 10.8

***Volumetric blood flow [pL/s]:***

control - 21.0 ± 5.5 31.7 ± 5.8 28.7 ± 3.5 26.3 ± 5.9

cryopreserved - 21.5 ± 5.7 23.9 ± 3.7 22.4 ± 3.0 22.1 ± 3.3

_________________________________________________________________________________________________

Mean ± SEM. No significant differences between the groups.
