# Supplementary figures and images for: Short-term cryoprotectant-free cryopreservation at −20°C does not affect the viability and regenerative capacity of nanofat
Source: Front Bioeng Biotechnol. 2024 Jul 1;12:1427232. doi: 10.3389/fbioe.2024.1427232 (PMC11246958; doi:10.3389/fbioe.2024.1427232)

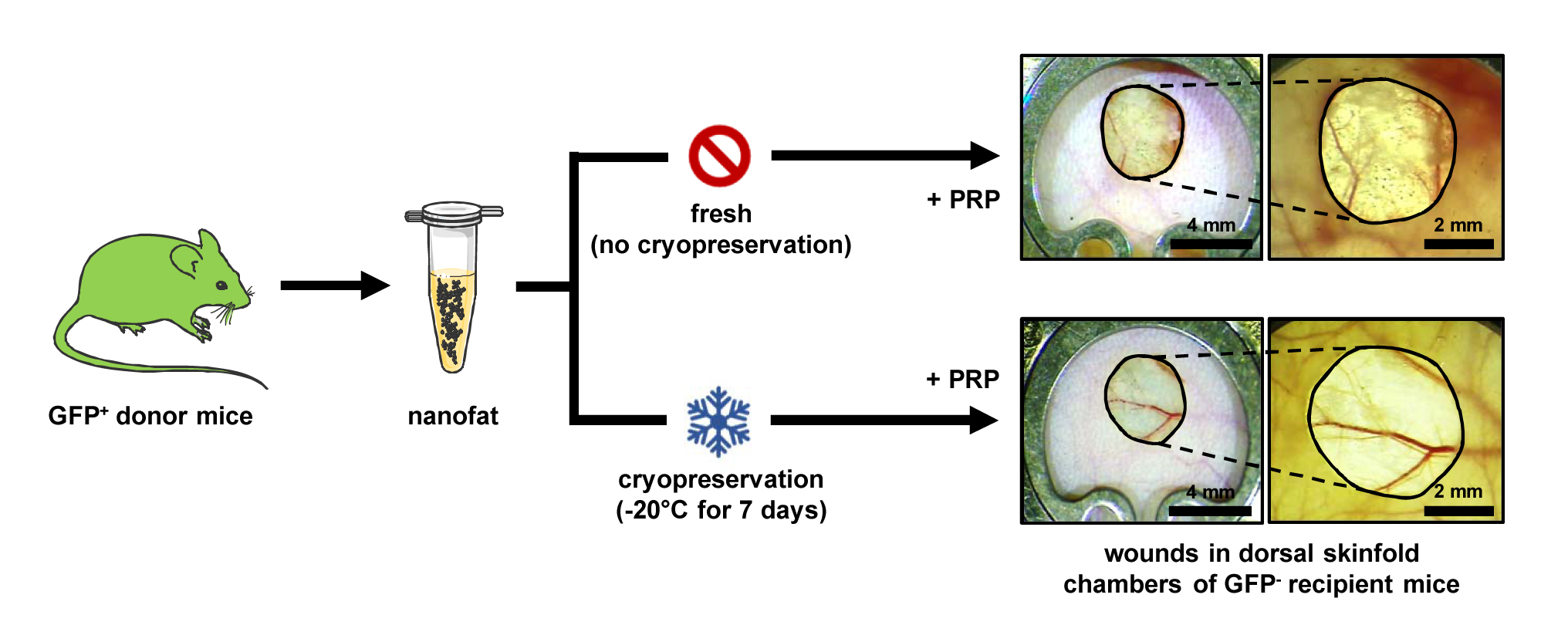

Supplement: Supplementary file 2 [file Image1.JPEG]

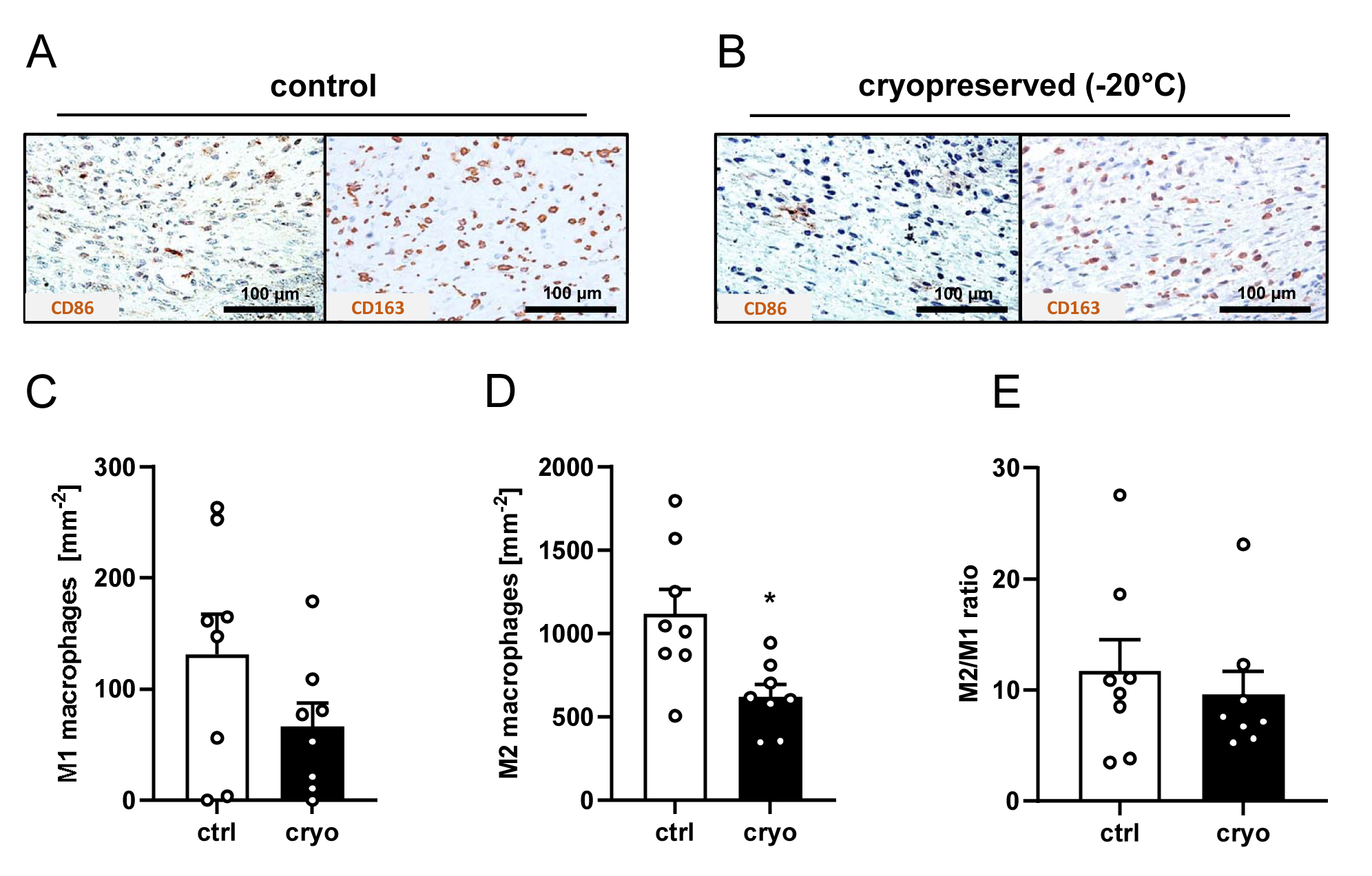

Supplement: Supplementary file 3 [file Image2.JPEG]
